# Evaluation of the Antimicrobial Activity of Geraniol and Selected Geraniol Transformation Products against Gram-Positive Bacteria

**DOI:** 10.3390/molecules29050950

**Published:** 2024-02-21

**Authors:** Anna Fajdek-Bieda, Joanna Pawlińska, Agnieszka Wróblewska, Agnieszka Łuś

**Affiliations:** 1 Department of Energy and Technical Safety, Faculty of Technology, Jakub’s from Paradyż Academy in Gorzów Wielkopolski, Teatralna 25, 66-400 Gorzów Wielkopolski, Poland; 2 Multispecialty Regional Hospital in Gorzow Wielkopolski, Department of Microbiology, 66-400 Gorzów Wielkopolski, Poland; joanna.pawlinska@szpital.gorzow.pl (J.P.); agnieszka.lus@szpital.gorzow.pl (A.Ł.); 3 Department of Catalytic and Sorbent Materials Engineering, Faculty of Chemical Technology and Engineering, West Pomeranian University of Technology in Szczecin, Piastów Ave. 42, 71-065 Szczecin, Poland

**Keywords:** *Staphylococcus epidermidis*, *Enterococcus* genus, terpenes, natural cosmetic

## Abstract

Both geraniol and the products of its transformation, thanks to their beneficial properties, find a variety of applications in cosmetics. Due to their antioxidant and moisturizing properties, these compounds can be added to skin care products such as face creams, lotions, oils, and masks. In addition, these compounds show some antibacterial and antifungal properties, making them suitable for application in skin care products to help fight against bacteria or fungi. This study determined the antimicrobial activity of geraniol and the compounds which were formed during its transformation in relation to selected Gram-positive bacteria, and the preliminary assessment was made whether these compounds can act as ingredients of preparations with potential antimicrobial activity in the treatment of various human diseases (for example diseases of the skin, digestive system, or urinary tract). In addition, this work presents studies on the microbiological purity of cream samples obtained with different contents of geraniol and its transformation products (contents of the tested compounds: 0.5%, 1.5%, 2.5%, 4%, 8%, and 12%). Antibacterial activity tests were performed using the disc diffusion method against Gram-positive cocci, including the reference strains *Staphylococcus aureus* ATCC 29213 and *Enterococcus faecalis* ATCC 29212, and against the clinical strains *Staphylococcus aureus* MRSA, *Staphylococcus epidermidis, Enterococcus faecalis* VRE VanB, *Enterococcus faecium* VRE VanA, and *Enterococcus faecium* VRE VanB. The most active ingredient against bacteria of the *Staphylococcus* genus was citral, followed by linalool and then geraniol. During our tests, in the case of bacteria of the *Enterococcus* genus, citral also showed the highest activity, but linalool, ocimenes, and geraniol showed a slightly lower activity. Moreover, this study examined the microbiological purity of cream samples obtained with various contents of geraniol and its transformation products. In the tests of the microbiological purity of cream samples, no growth of aerobic bacteria and fungi was found, which proves the lack of microbiological contamination of the obtained cosmetic preparations. On this basis, it was assessed that these compounds have preservative properties in the prepared creams. The addition of the analyzed compounds also had influence on the durability of the creams and had no effect on the change in their consistency, did not negatively affect the separation of phases during storage, and even had a positive effect on organoleptic sensations by enriching the smell of the tested samples.

## 1. Introduction

An important issue related to cosmetic products is their hygienic cleanliness. Many ingredients used to produce cosmetics can create favorable conditions for the development of bacteria and fungi. These microorganisms can significantly affect the quality of the cosmetic product and it can even result in its rejection. Changes in appearance, color, unpleasant smell, separation of ingredients, precipitation of sediments, or changes in consistency caused by the activity of microorganisms significantly affect the experience of using the cosmetic. To effectively prevent the growth of microorganisms, it is necessary to use appropriate preservatives. The effective preservative should be highly active against various microorganisms, even in small amounts, and should also be effective over a wide pH range. Moreover, it should be water-soluble, non-toxic, and should not cause irritation or allergies. However, it is worth remembering that some preservatives may cause allergic reactions, which is why their amount in cosmetic products, especially those intended for oral hygiene, is minimized [1].

In the case of natural cosmetics, also known as phytocosmetics, it is necessary to use natural preservatives. For this purpose, essential oils are often used, which effectively inhibit the development of microorganisms. While serving as preservatives, essential oils also have the ability to serve other important roles, such as fragrances or active ingredients. In this way, in addition to preserving the durability of cosmetic products, they can give them therapeutic or caring properties, which further increases their value. The main components of essential oils, i.e., terpenes, are promising substances in cosmetic products due to their relatively safe nature. Due to the variety of biological activities, essential oils are valued as desirable ingredients in cosmetics [2].

In recent years, there has been a visible increase in the interest in products of natural origin in the food, pharmaceutical, and cosmetics sectors, as well as in medicine. Consumers’ greater awareness of the destructive impact of the environment and free radicals on human health makes us more willing to reach for food products, cosmetics, and drugs based on natural plant raw materials, because we are convinced of their health-promoting effects [1]. In highly developed countries, the “return to nature” is particularly intensely observed in the pharmaceutical and cosmetic industries, which may result from growing concerns about the environment, as well as from the new needs and interests of modern society, referring, among others, to ethical values and animal rights. Highlighting the problem of the eco-friendliness of cosmetic products makes consumers more willing to buy more expensive products that are of high quality and, at the same time, contain ingredients of plant origin, because they consider them safer. This, in turn, drives the market and forces producers to create new product groups [2].

By definition, a “natural cosmetic” is a product that is intended to beautify and nurture using natural substances, be friendly to the skin and the natural environment, promote health, support the body’s self-regulation, and support the long-term maintenance of natural beauty and the harmonious development of the body and spirit. The product should be composed of ingredients of natural origin (mainly plant and mineral origin), obtained using physical methods (e.g., pressing, extraction, filtration, distillation, drying, etc.), microbiological methods, or enzymatic methods. The ingredients mentioned above include various types of oils, extracts, and hydrolates [2,3]. Natural cosmetics may also contain ingredients of animal origin, e.g., honey or beeswax, provided that the production process is animal friendly. Products can be labelled as a “natural cosmetic” when the product contains at least 95% natural ingredients and the remaining 5% are synthetic ingredients, including safe preservatives, approved for use in food [3,4].

Unfortunately, many cosmetics currently available on the market contain formaldehydes or parabens, petroleum derivatives, PEG emulsifiers, or detergents such as SLS or SLES, which have skin-drying properties, as well as containing synthetic antioxidants as preservatives [5]. It is known that oxidation reactions can cause the formation of free radicals, and an increase in the amount of free radicals can initiate the chain reactions which are responsible for skin aging processes, such as the loss of elasticity, as well as the appearance of discolorations and wrinkles [5,6]. On the other hand, antioxidants are used in cosmetics to extend the shelf life of products. Moreover, they act as bioactive cosmetic ingredients that can protect the skin against the effects of free radicals by the inhibition of the oxidation reaction [6]. Therefore, the production of cosmetics, including natural ones, requires the use of appropriate preservatives. This gives the cosmetic product’s formula stability and protects them against changes in composition, consistency, smell, and bacterial multiplication. Every consumer expects that the cosmetic products that they use are safe and also that they will not change their appearance, smell, or consistency during use [7,8]. Due to the fact that the production of cosmetic preparations uses water, as well as many raw materials and components that can constitute the ideal environment for the reproduction of microorganisms, a very important issue regarding cosmetics is their microbiological purity [9].

The microbiological contamination of cosmetics constitutes a significant threat to the quality of the product, as it may cause changes in the consistency of the preparation, it can also cause a color change precipitation, as well as changes in the organoleptic and aesthetic properties. These changes often exclude the product from further application. However, the greatest threat related to the contamination of cosmetics with pathogenic microflora is the loss of the therapeutic and care properties of cosmetic preparations and even the loss of consumers’ health. Therefore, the production of safe care products, regardless of whether they are natural cosmetics or not, includes the use of preservative systems to protect them against the development of microorganisms during the process of production, as well as during their storage and use. Bacteria, yeasts, and molds can get into the product after opening the package, through contact with a finger during application or even through contact with air [3]. It is important that the selected preservatives are safe for the consumer and, at the same time, inactivate a wide spectrum of microbiological contaminants (Gram-positive and Gram-negative bacteria, yeasts, and molds). They should also be compatible with the formula and stable throughout the product’s shelf life, whilst also meeting the applicable legal standards and being consistent with the ever-changing market needs. However, the synthetic preservatives and antioxidants commonly used in cosmetics may cause allergic reactions, irritations, and even excessive drying of the skin [2,3].

The emerging controversies surrounding the use of certain preservatives in cosmetic products and the growing demand for natural cosmetics make it necessary to look for their new, ecological equivalents that do not cause negative effects on users. This gave rise to the idea of replacing the synthetic preservatives with essential oils, which have a wide range of biological properties, including antimicrobial, anticancer, and antioxidant properties [8,9]. Moreover, the essential oils contained in plants have a broad inhibitory effect against various Gram-negative and Gram-positive bacteria. However, it is worth paying attention to the fact that most essential oils will have a stronger effect on Gram-positive bacteria than on Gram-negative bacterial species, which is related to differences in the composition of their cell membrane. Gram-negative bacteria have a more complex cell wall structure (LPS (side chain O and lipid A), lipoprotein, phospholipids, and outer membrane), which makes it difficult to transport hydrophobic molecules, such as the organic compounds present in essential oils (mainly terpenes) [10]. Moreover, studies have shown that some essential oils have killing properties against drug-resistant bacteria [11,12] and also have the potential to have anti-biofilm and anti-adhesive activity [13].

The mechanism of action of the essential oils depends on their chemical composition, while their antimicrobial activity is not associated with a unique mechanism, but is instead associated with the occurrence of a cascade of reactions involving the entire bacterial cell. Interestingly, the action of the oils includes not only the inhibition of the growth of bacterial cells, but also the inhibition of the production of toxic bacterial metabolites [12,13]. It is worth adding that an important advantage of essential oils is that they are usually easily available and contain a large number of various organic compounds. Mixtures of essential oils can be used to support the preservation of cosmetic preparations, and, additionally, if properly selected, they can act as a fragrance and extend the life of cosmetics. The main components of essential oils—terpenes—deserve special attention. Terpenes and their derivatives constitute a significant group of natural organic compounds that occur in numerous essential oils [14]. Extensive research has confirmed that terpene ingredients have extensive biological properties in in vitro tests. It was found that they have, among others, antibacterial, antiviral, antifungal, and antiparasitic abilities. Moreover, they also show an anti-inflammatory effect and can stimulate the immune system [13].

**Figure 1:**
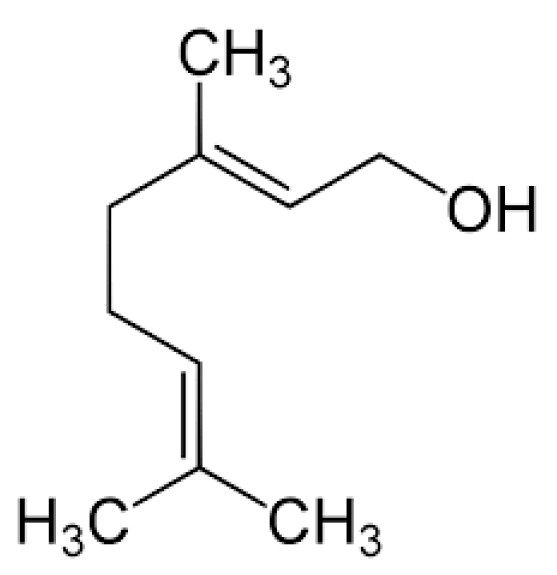
Structure of geraniol.

Geraniol (Figure 1) is the terpene alcohol present in the essential oils of many plants with a characteristic rose scent. Geraniol has been used, among others, in the perfume industry as the component of many fragrance mixtures [15].

The properties of geraniol include repellent and insecticidal properties, which make it useful as a natural pest control agent, while maintaining a low toxicity to mammals [16]. Moreover, geraniol has the ability to combat the *Tyrophagus putrescentiae* mite and has a strong antiparasitic effect against the root node *Meloidogyne incognita* [17]. Research also confirms that geraniol belongs to a new group of chemopreventive compounds that can be used in the fight against various types of cancer, such as murine leukemia, melanoma, and colon cancer [18]. Geraniol also has antioxidant and anti-inflammatory properties, as well as antimicrobial activity—this compound is active against the bacteria *Listeria monocytogenes* (BA50 0.28) and *Salmonella enterica* (BA50 0.15) [19]. Additionally, geraniol in a gaseous form has the ability to fight with respiratory pathogens, including *Haemophilus influenzae*, *Streptococcus pneumoniae*, *Streptococcus pyogenes*, and *Staphylococcus aureus*. Studies have also demonstrated the anti-tuberculin activity of geraniol against *Mycobacterium tuberculosis* [20]. Other studies have confirmed that palmarosa oil, whose antifungal activity is mainly attributed to geraniol, is effective against *Candida albicans* [21]. Geraniol also limits the development of the biofilm of this fungus and inhibits the development of *Cryptococcus neoformans*, the pathogen associated with the infections in the advanced phase of AIDS [22]. Analysis of the antioxidant activity of geraniol, in comparison to a standard antioxidant (Trolox), shows that geraniol has the ability to neutralize the DPPH radical at the level of 87.7% (equivalent to 235.9 mg Trolox/mL) [23]. Additionally, studies examining the immunosuppressive properties of geraniol have shown that it can effectively prevent acute heart transplant rejection in a rat allograft model [24]. Transdermal drug delivery systems, which are now increasingly used, are an innovative method of therapy, and geraniol can be used to increase drug penetration in such systems. Studies have shown that the addition of tetrahydrogeraniol to a gel containing 5-fluorouracil significantly increased the penetration capacity of this compound [25,26].

**Figure 2:**
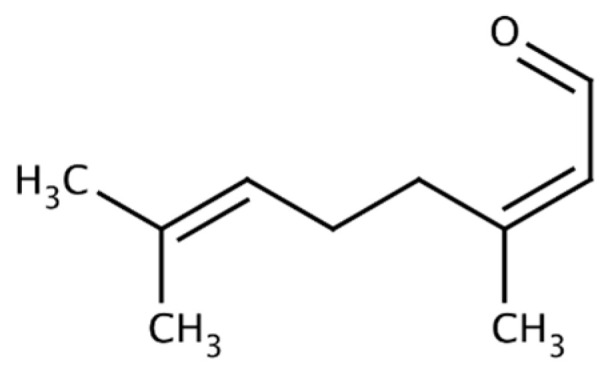
Structure of citral.

Citral (Figure 2), a compound also belonging to the terpene family, is distinguished by its distinct lemon scent. This compound is the component of many essential oils, including lemon oil, lemongrass oil (80% citral), and oils obtained from tropical plants, such as *Backhousia citriodora* (lemon myrtle, 90% citral), lemon verbena (30–35% citral), lemon balm, and orange [27].

Citral is used in the perfume and food industries as a fragrance ingredient and flavor enhancer [28]. Studies have shown that this compound has antiparasitic, antibacterial, antioxidant, and anti-inflammatory properties [29]. Moreover, in vitro studies conducted with citral confirmed its anticancer potential. Citral inhibits the growth of the human breast cancer cell line MCF-7, and has a beneficial effect in B-cell lymphoma patients treated with chemotherapy. Additionally, it limits the viability, proliferation, and clonogenic capacity of prostate cancer cells [30]. Citral can also be used as an effective painkiller [31]. Orally administered citral shows a significant protection of the stomach against ulcers caused by indomethacin, which acts as an anti-inflammatory drug [32]. Studies also demonstrate the ability of citral to protect IEC-6 cells from oxidative stress caused by aspirin [33]. Espina et al. [34] examined the effects of citral and carvacrol on the bacteria *L. monocytogenes* (EGD-e), *S. aureus* (SC-01), and *E. coli* (MG1655). These studies showed that both citral and carvacrol caused a decrease in the number of bacterial cells and reduced the formation of biofilms by all three species. Moreover, the tests showed that α-citral and β-citral have an antibacterial activity against both Gram-positive and Gram-negative bacteria. The experiments also focused on assessing the antimicrobial effect of citral against *Yersinia enterocolitica*. The minimum inhibitory concentration and minimum bactericidal concentration of citral for the *Y. enterocolitica* ATCC 23715 strain were 0.2 and 0.4 mg/mL, respectively. Citral also has the ability to inhibit the growth of the fungus *Candida albicans* [35,36,37,38,39]. Cai et al. [40] also conducted tests in relation to the antifungal activity of citral, limonene, and eugenol against *Zygosaccharomyces rouxii*. All three compounds showed a strong antifungal activity against *Z. rouxii*. The minimum inhibitory concentrations of citral, limonene, and eugenol in this study were 0.188, 0.75, and 0.4 µL/mL, respectively, and the minimum fungicidal concentrations of these compounds were 0.375, 3, and 0.8 µL/mL, respectively [40].

**Figure 3:**
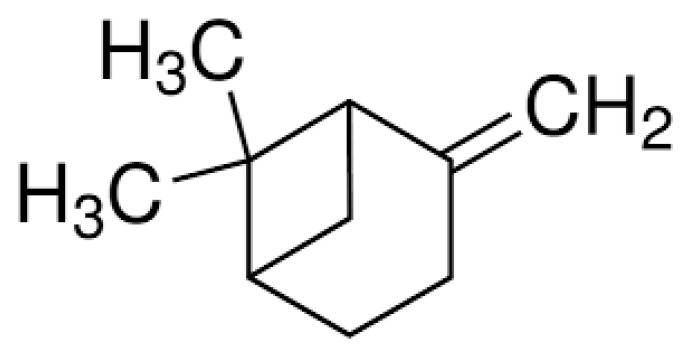
Structure of beta-pinene.

Beta-pinene (β-pinene) (Figure 3) is another compound obtained as a result of the isomerization process of geraniol, which also has a proven antimicrobial activity [41].

Studies reported in the scientific literature assessed the effectiveness of eugenol, beta-pinene, and alpha-pinene in suppressing the growth of Gram-positive bacteria that can lead to endocarditis. The minimum inhibitory concentrations (MICs) for these phytochemicals were determined using the solid support diffusion procedure. The effect of MIC values on bacterial cell survival was then investigated by assessing the number of viable microorganisms. *S. aureus*, *S. epidermidis*, *Streptococcus pneumoniae*, and *Streptococcus pyogenes* strains were used as the test microorganisms. The experiments showed that the tested phytochemicals effectively inhibited the growth of all analyzed bacterial strains, with MIC values ranging from 2.5 to 40 µL/mL. It is worth emphasizing that eugenol showed the lowest MIC values, ranging from 2.5 to 5 µL/mL for most of the tested bacterial strains. Furthermore, test results showed that the MIC values of the phytochemicals were sufficient to completely eliminate the *S. aureus* bacterial inoculum within a maximum of 24 h of exposure. These results provided important information about the promising antibacterial properties of the analyzed phytochemicals and their potential applications in antimicrobial therapy [42].

Other studies reported in the scientific literature assessed the antimicrobial activity of pinene isomers and enantiomers against bacterial and fungal cells. The agar diffusion test showed that only the positive enantiomers of α- and β-pinene isomers showed activity. The minimum inhibitory concentration (MIC) and minimum microbicidal concentration (MMC) of these monoterpenes were also determined, confirming that the positive enantiomers exhibited a microbicidal activity against all fungi and bacteria tested, with MICs ranging from 1.17 to 4.150 µg/mL. However, no antimicrobial activity was found using the negative enantiomers, even up to 20 mg/mL. Killing activity–time curves against the tested strains showed that (+)-α-pinene and (+)-β-pinene were highly toxic to *C. albicans*, killing 100% of fungal cells within 60 min. However, the bactericidal effect occurred only after 6 h in the case of *S. aureus* (MRSA). Moreover, ciprofloxacin together with (+)-α-pinene or (+)-β-pinene showed a synergistic effect against *S. aureus* MRSA, while the use of amphotericin B in combination with the positive pinene enantiomers showed a neutral effect against all fungi. The potential of (+)-α-pinene and (+)-β-pinene to inhibit phospholipase and esterase activities was also evaluated, and the best inhibition results were obtained with *Cryptococcus neoformans*. Biofilm formation by *C. albicans* was prevented at MICs of (+)-α-pinene and twice the MIC of (+)-β-pinene [43,44,45,46].

**Figure 4:**
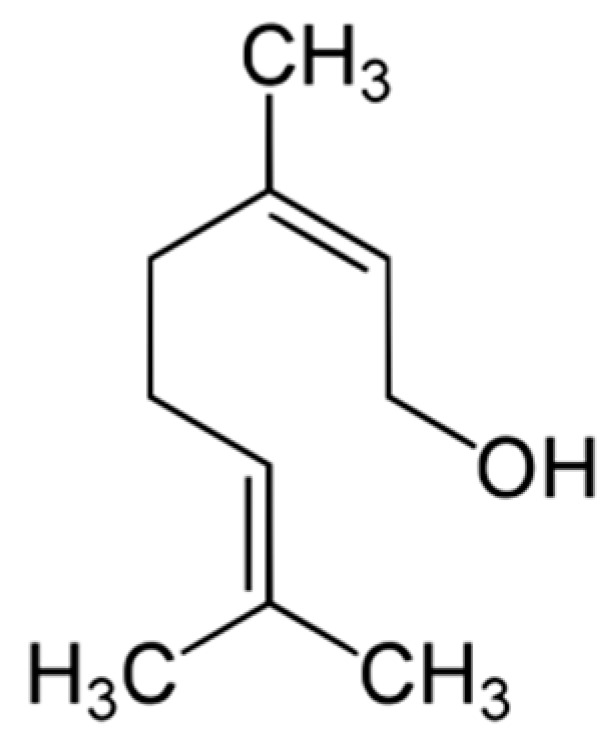
Structure of nerol.

Nerol (Figure 4) is another compound obtained by the isomerization of geraniol. It is characterized by a delicate rose scent, which is more pleasant and subtle than the smell of geraniol, of which it is the geometric isomer. Nerol naturally occurs in bergamot and neroli oils [47].

Nerol is a more valuable ingredient in fragrance compositions than geraniol, and it is used in higher quality fragrance compositions and in luxury products [48]. Moreover, it is an ingredient of decorative cosmetics, shampoos, and soaps, and it is also found in other cleaning products and detergents [49]. Research shows that neroli oil has significant healing properties [50]. Among other things, it has an antiseptic effect and is used in compresses to relieve the symptoms of mycosis and skin inflammation [51]. Due to its antidepressant properties, neroli oil is popular in aromatherapy—it helps improve mental health, reduces stress, relieves anxiety, and strengthens the mind. Used in massage, it supports the condition of capillaries, improves blood circulation, and relaxes muscles [52]. Neroli oil also helps alleviate the symptoms of menopausal syndrome and premenstrual syndrome [53]. The literature describes studies conducted on mice investigating the neuropharmacological properties of nerol. In the context of antimicrobial activity, nerol showed activity against various species of bacteria (both Gram-positive and Gram-negative), yeast, and mold fungi. It was particularly effective in combating Gram-negative bacteria, such as *P. aeruginosa*. In the disc diffusion method test, nerol showed stronger antifungal properties compared to a standard antifungal antibiotic (nystatin) [54].

**Figure 5:**
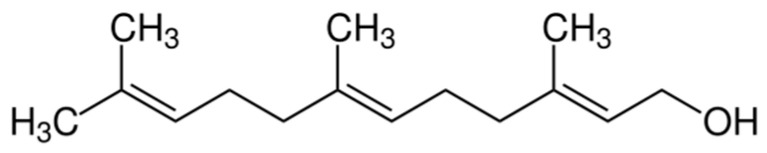
Structure of farnesol.

Farnesol (Figure 5) is a sesquiterpene alcohol produced by many organisms and is present in several essential oils [55].

The role of farnesol as a signaling molecule and as a factor influencing the virulence of *Candida albicans* has been thoroughly investigated and described in the literature [56]. The results of studies described in the scientific literature indicate that farnesol affects the growth of numerous bacteria and fungi, which suggests the possibility of using it as a potential antimicrobial agent. In order to investigate the effect of farnesol on the growth of *Paracoccidioides brasiliensis* cells, tests were performed measuring the optical density of the cultures [57]. The viability of these fungal cells was assessed by both the MTT assay and by counting colony-forming units after each farnesol treatment. Additionally, the effect of farnesol on the dimorphism of *P. brasiliensis* was examined using the method of optical microscopy. The ultrastructural structure of *P. brasiliensis* yeast cells treated with farnesol was analyzed using transmission and scanning microscopy. This study presents the effects of farnesol on the growth and dimorphism of *P. brasiliensis*. Concentrations of this isoprenoid ranging from 25 to 300 μM significantly inhibited the growth of *P. brasiliensis*. The minimum inhibitory concentration (MIC) of farnesol for *P. brasiliensis* was determined to be 25 μM, while the minimum killing concentration (MBC) was found to be around 30 μM. When using farnesol concentrations from 5 to 15 μM, which did not affect the viability of the fungus, an effect on the morphogenesis of this fungus was also observed. A concentration of 15 μM farnesol caused an approximately 60% inhibition of hyphal formation by *P. brasiliensis* yeast cells for a period of 48 h. At these farnesol concentrations, a significant shortening of the mycelial hyphae was also observed. Electron microscopy studies showed that, despite maintaining an intact cell wall, *P. brasiliensis* cells treated with farnesol concentrations above 25 μM showed a complete cytoplasmic degeneration. Taken together, the results indicate that farnesol acts as a potent antimicrobial agent against *P. brasiliensis*. The fungicidal activity of farnesol against this pathogen is probably related to cytoplasmic degeneration. Concentrations of farnesol that do not negatively affect the viability of the fungus delay the formation of *P. brasiliensis* hyphae, suggesting that the morphogenesis of this fungus is controlled by the environmental conditions [57]. *Staphylococcus aureus* is one of the main pathogens responsible for sepsis and is also capable of forming biofilms on the host tissues and implanted medical devices, thereby causing systemic infections within the host [58]. Infections caused by *S. aureus* are becoming increasingly difficult to treat due to the increasing resistance of this bacterium to antibiotics. Especially in the biofilm environment, microorganisms show an increased resistance to antimicrobial agents. Recently, scientific research has shown that farnesol has been classified as a signaling molecule with potential antimicrobial properties. The above-mentioned study examined the effect of farnesol on methicillin-resistant and sensitive *S. aureus* strains. Through viability tests, assessment of biofilm formation, and ethidium bromide absorption studies, farnesol has been shown to inhibit biofilm formation and to destroy the integrity of the cell membranes. The effect of farnesol on the increasing antimicrobial susceptibility of *S. aureus* was assessed using the agar diffusion and broth microdilution tests. For both types of staphylococci, farnesol was able to reverse the resistance only at a high concentration (150 μM). However, it effectively increased the effect of antibiotics to which the strains were, to some extent, susceptible. Therefore, studies relating to the synergy of farnesol and gentamicin were performed on static biofilms, exposing them to different concentrations of both compounds. Counting of the biofilm cells sampled at 0, 4, and 24 h after the treatment showed that the cumulative effect of gentamicin at 2.5 times the MIC and farnesol at the concentration of 100 μM (22 μg/mL) was able to reduce the bacterial population by more than 2 log units, which proves the synergy between both antimicrobial substances. The observed increase in the sensitivity of resistant strains to antibiotics and the observed synergy with gentamicin suggest the potential application of farnesol as an additional therapeutic agent in the prevention of biofilm-related infections and in the reversal of drug resistance [58,59].

**Figure 6:**
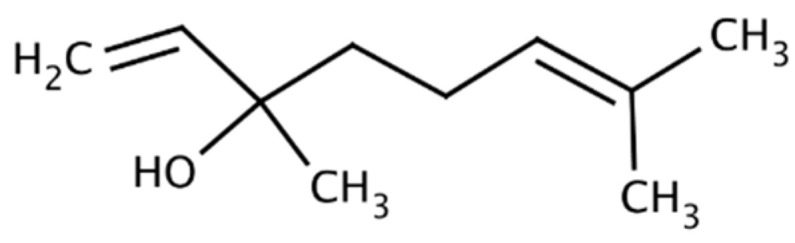
Structure of linalool.

Linalool (Figure 6), an aliphatic alcohol from the terpene group, has a delicate scent reminiscent of lily of the valley. Its natural source is essential oils, e.g., coriander or orange. In the cosmetic and perfume industries, linalool is commonly used as a fragrance ingredient [60].

Linalool, together with essential oils rich in this compound, shows various biological effects (antibacterial, anti-inflammatory, anticancer, and antioxidant) [61]. At a concentration of 0.1% (*v*/*v*), linalool shows antimicrobial activity against various microorganisms, such as *S. aureus*, *Bacillus subtilis*, *E. coli*, and *Pasteurella multocida*. It has a particularly positive effect on Gram-positive bacteria in comparison to Gram-negative bacteria [62,63]. Moreover, linalool has the unique ability to fight periodontopathic and cariogenic bacteria. Its minimum inhibitory and bactericidal concentrations range from 0.1 to 1.6 mg/mL. It is worth emphasizing that linalool, which was obtained from coriander, has an antifungal activity against *Candida* isolates in the oral cavity of patients with dental problems, as well as against the various clinical isolates of *C. albicans* and *Candida non-albicans* (NAC) with varying degrees of sensitivity to fluconazole [64]. In various experimental models of inflammation, both forms of the linalool enantiomer and its racemate have anti-edematous effects, limiting the inflammatory response. It is worth adding that linalool has antiproliferative effects on many types of cancer cells, including those resistant to classical treatment. In the case of malignant hematopoietic tumors, linalool does not affect the development of healthy hematopoietic cells, even at its cytotoxic concentration of 130 µM. However, it should be noted that its anticancer potential in in vivo tests is limited due to the need to use high doses. In the field of aromatherapy, specialists use linalool to relieve anxiety symptoms, and the anxiolytic properties of this compound have been confirmed in many studies. Mice treated with linalool showed less aggressiveness and anxiety, which improved their social interactions [65]. Recent research on linalool has focused on its potential use in the treatment of Alzheimer’s disease in a mouse model. It was found that mice treated with linalool showed improved cognitive and emotional functions [66]. Moreover, in agriculture, linalool is used as a fumigant, i.e., a substance that repels pests [65].

**Figure 7:**
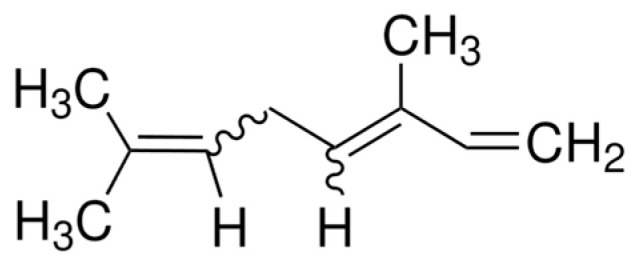
Structure of ocimene.

Ocimenes (Figure 7) are found in a variety of plant oils, including marigold and lavender oils. Ocimenes exhibit strong antibacterial properties. They are capable of combating various bacteria and viruses, making them often used in the treatment of respiratory infections and issues related to bacterial infections [67].

Compounds present in ocimenes possess anti-inflammatory properties, contributing to the alleviation of inflammatory conditions in the body [68]. They contribute to the benefit of the immune system, aiding in the fight against infections and strengthening the body’s defense [69]. Ocimenes also demonstrate antioxidant activity, helping to combat free radicals and protect cells from oxidative stress [70]. Oregano, containing ocimenes, can be applied to alleviate digestive discomforts such as indigestion and bloating. Ocimene oils are commonly used for inhalation to ease symptoms of colds, congestion, cough, and upper respiratory infections [71]. The external application of ocimene oils can be beneficial for minor skin problems, such as insect bites or mild irritations [72].

**Figure 8:**
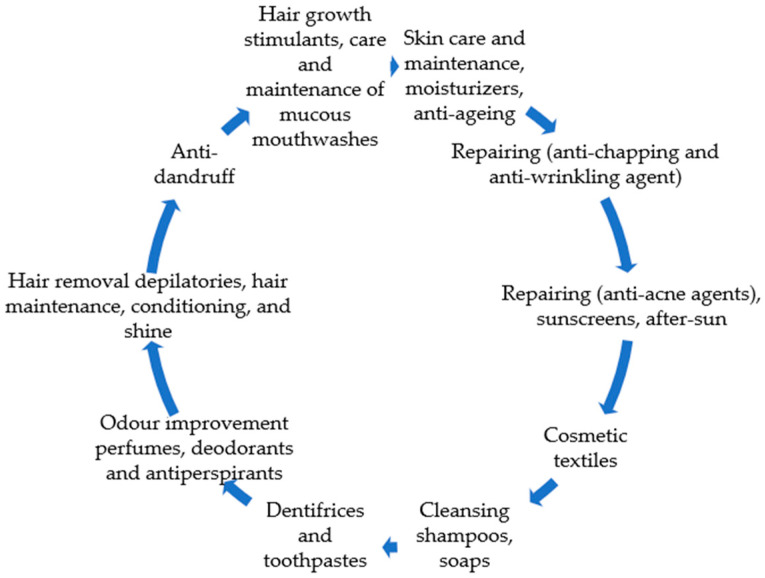
Example of essential oils present in the different categories of cosmetic products.

Essential oils are present in various types of cosmetic products and perform very different functions. This is generally presented in Figure 8. Essential oils are added to products of natural origin, among other things, to give them an appropriate scent. The most popular oils added for this purpose include oils from rose, tuberose, narcissus, gardenia, jasmine, and lavender [58], as well as oils from patchouli, citronella, sandalwood, bergamot, rosemary, mint, and vetiver [73].

Studies published in the scientific literature by Lubbe et al. [74] showed that essential oils with antibacterial properties, such as tea tree oil (*Tea tree*) and peppermint oil (*Mentha piperita*), effectively prevent the growth of microorganisms in cosmetic formulations containing the collagen hydrolyzate. The antibacterial effectiveness of these formulations was tested on *Staphylococcus aureus*, *Escherichia coli*, *P. aeruginosa*, *Enterococcus faecalis*, *Aspergillus fumigatus*, and *Candida albicans*. These studies showed that formulations containing 2% essential oils had the highest antibacterial activity, and both oils were effective against such microorganisms as *S. aureus*, *E. coli*, *A. fumigatus*, and *C. albicans*. The control formulation did not show any antibacterial or antifungal effects. These studies have shown that the use of appropriately selected essential oils in moderately low concentrations prevents the development of pathogenic microorganisms in cosmetic products [74]. In the case of oral care products, essential oils also show similar effectiveness. Research conducted by Adwan et al. [75] showed that mouthwashes containing selected essential oils (thymol, menthol, and eucalyptol) have a similar effect against the bacteria causing plaque formation as commercial mouthwashes containing alcohol. The essential oils used in these studies also offer additional benefits, such as the reduction of gum inflammation and improving breath freshness. These results indicate that essential oils may successfully replace synthetic chemicals in the future, which are often used in cosmetics as preservatives and antibacterial agents but are also harmful to health. Additionally, essential oils can be used as cooling agents. For example, peppermint and eucalyptus oils leave a long-lasting feeling of refreshment on the skin and in the mouth [46].

Many studies have shown [35,62] that essential oils are an excellent source of natural antioxidants. Examples of sources of substances with a strong antioxidant effect include essential oils from the species *Clausenaanisata* and *Eucalyptus camaldulensis* [62], as well as essential oils obtained from rosemary [35], coriander, eucalyptus, juniper, cumin, basil, cinnamon, cloves, thyme, and Egyptian poppy [76].

Essential oils are also used in hair care products, adding a shine and conditioning effect, as well as protecting and strengthening the scalp. Chamomile and rosemary oils, and also bay oil from the Caribbean help to condition and improve hair growth [77]. Bergamot and tea tree oils can help fight dandruff [78]. Moreover, nowadays, essential oils are also used in cosmetic products with cosmeceutical properties. An example is geranium oil, which is used in cleansing cosmetics for oily skin and in cases of acne and eczema problems [79]. Oils from the species *Agathosma betulina*, *Eriocephalus africanus*, and *Eriocephalus punctulatus* also have anti-inflammatory properties [80]. Chamomile oil also has anti-inflammatory properties and it is used in the treatment of skin inflammation [81,82].

Essential oils contain numerous biologically active compounds, which makes them very attractive for both medicines and cosmetics. However, essential oils are not fully used in medicines and cosmetics. One of the problems may be the uniqueness of their composition and a lack of complete information about the biological activity of all ingredients, including their toxic effect on the body. The solution may be to isolate the most important components from the oils, purify them, and use them as single ingredients in cosmetic and drug formulations. After purification, these ingredients can also be subjected to isomerization and oxidation processes, thanks to which it will be possible to obtain ingredients that can demonstrate a higher biological activity than the raw materials from which they were formed. In recent years, an increasing drug resistance (antibiotic resistance of many bacteria) has been observed. The solution to this problem may be terpene compounds obtained from essential oils and their derivatives obtained from simple chemical reactions carried out using heterogeneous catalysts. Such compounds of natural origin can also be very effective in fighting many bacterial infections, e.g., limonene or α-pinene, which are successfully used in the treatment of respiratory infections. An additional advantage of terpenes is their renewability (they are obtained from plant biomass and, in some cases, also from waste plant mass) and environmental friendliness. Catalytic isomerization and oxidation processes carried out using heterogeneous catalysts are very cheap and easy to conduct, and do not often require the use of a solvent or complicated equipment. The catalysts used in these processes can be easily separated from the post-reaction mixture and regenerated. Additionally, it should be emphasized that these catalysts are often minerals (porous materials of natural origin, which are relatively cheap and easily available), which is also in line with current trends in chemical technologies, including methods of preparation of raw materials for cosmetics and medicines. These technologies should meet the requirements of sustainable development and be environmentally friendly. Such technologies include technologies for obtaining raw materials for cosmetics and medicines, using terpenes and minerals of natural origin.

The aim of our work was as follows:

(1) The determination of the antimicrobial activity of geraniol and compounds formed during the transformation of geraniol in relation to selected Gram-positive bacteria (the research concerned pure, individually used compounds) and a preliminary assessment as to whether these compounds can act as ingredients of preparations with potential antimicrobial effects in the treatment of various diseases (skin diseases, digestive system diseases, respiratory system diseases, arthritis, urinary tract infections, and infections of postoperative and post-burn wounds).
(2) The examination of the microbiological purity of cream samples obtained with various contents of geraniol and its transformation products (content of tested compounds: 0.5%, 1%, 2.5%, 4%, 8%, and 12%) and an assessment, based on the results obtained, as to whether these compounds show preservative properties in prepared cream samples, thereby reducing the risk of the development of microorganisms, causing a reduction in the quality of cosmetic products.

## 2. Results and Discussion

### 2.1. Antibacterial Activity of Geraniol and Products of Its Transformation

This study tested the antibacterial activity of geraniol and selected products of its transformation (citral, β-pinene, nerol, farnesol, linalool, and ocimenes), which are components of many valuable essential oils with confirmed biological activity. Therefore, seven strains of Gram-positive bacteria were selected for testing. The results of the antibacterial activity of geraniol and selected products of its transformation against Gram-positive bacteria are summarized in Table 1. The activity of the tested compounds was as follows: citral, linalool > nerol > geraniol > ocimenes > farnesol > β-pinene. The tested strains were divided into two groups according to the type of *S.* spp. and *E.* spp.

**Figure 9:**
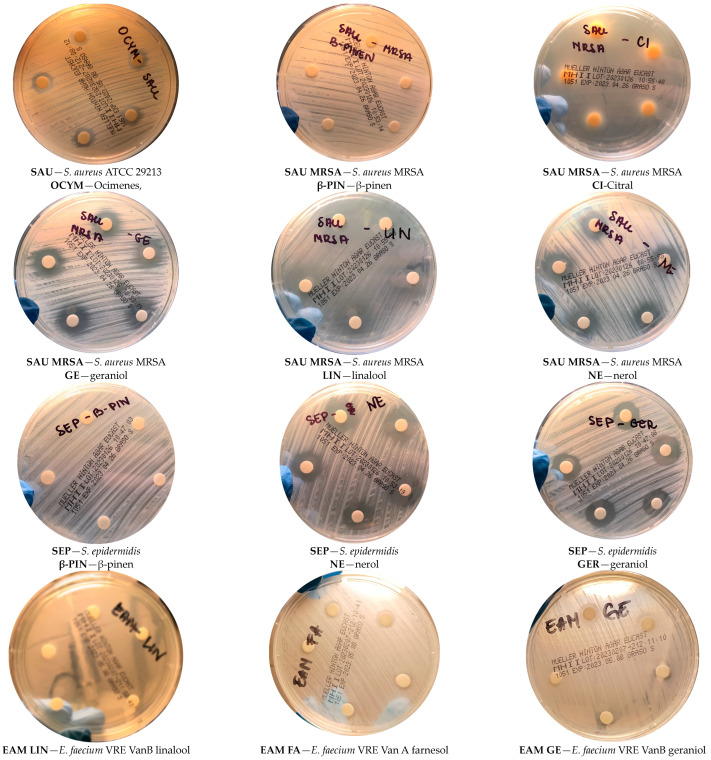
Sample photos of the results of the disc diffusion method.

The tested compounds showed the highest activity in the staphylococcal group against the *S. epidermidis* strain, respectively: GA 17.4 mm, CI 42.6 mm, BP 6 mm, NE 18.6 mm, FA 6 mm, LI 30 mm, and OC 10 mm. Slightly lower activities were obtained for the two remaining *S. aureus* strains, both clinical and reference strains. Only BP and FA did not show activity against all staphylococci (Figure 9). Particularly noteworthy are the sizes of growth inhibition zones obtained for LI (*S. aureus* ATCC 29213 40 mm, *S. aureus* MRSA 23.6 mm, *S. epidermidis* 30 mm) and CI (*S. aureus* ATCC 29213 39.6 mm, *S. aureus* MRSA 20 mm, *S. epidermidis* 42.6 mm). 

The second group of bacteria tested were *E.* spp. (Table 1), for which, similarly to *S.* spp., LI and CI had the highest activity, while BP and FA had the weakest activity. GE showed the strongest inhibitory effect on the growth of the bacteria *E. faecium* VRE VanA (20.2 mm), while the weakest effect was shown for *E. faecalis* ATCC 29212 (6 mm). For the remaining strains tested, the values were within the range of 9.8 mm for *E. faecalis* VRE VanB and 12 mm for *E. faecium* VRE VanB. CI showed more than twice the activity against *E. faecalis* ATCC 29212 compared to the clinical strain *E. faecalis* VRE VanB. In the case of the other two strains, this compound showed similar activity. In the tested group of strains, both NE and FA showed stronger properties inhibiting the growth of *E. faecium* strains compared to *E. faecalis* strains. Moreover, LI showed a similar antibacterial activity in the case of *E. faecalis* strains. Twice as high an activity for this compound was observed for the *E. faecium* strain with VRE VanB resistance than for the *Enterococcus faecium* strain with VRE VanA resistance. In the case of OC, the highest activity was obtained against the reference strain *E. faecalis* ATCC 29212 in relation to other enterococci (Figure 9).

Our studies showed that most of the tested compounds inhibited the growth of the tested strains of *Staphylococcus* bacteria. The most active compound against bacteria of the *Staphylococcus* genus was citral, followed by linalool and then geraniol. *S. aureus* bacteria have the ability to produce various toxins that may be responsible for specific diseases, including for staphylococcal food poisoning, toxic shock syndrome (TSS), and also for scalded skin syndrome (SSS), known as Ritter’s disease, which includes skin diseases of varying severity caused by toxins called exfoliatins [83]. These toxins cause the formation of blisters with thin walls that burst easily, as well as the creeping of the epidermis, leading to exposure of the dermis, which, in turn, leads to secondary superinfections that are difficult to treat [83]. It is worth mentioning that the most common infections caused by *S. aureus* include the purulent inflammation of the skin and soft tissues: boils, styes, impetigo, abscesses, phlegmons, septic arthritis, and endocarditis [84]. Multidrug-resistant staphylococci *S. aureus* (MRSA) and *S. epidermidis* (MRSE) are most often isolated from blood as the cause of hospital sepsis, as well as from postoperative wounds and implants, where they form a biofilm that is difficult to treat [84]. Our research results regarding the *Staphylococcus* bacterial strains listed in Table 1 indicate that geraniol and its derivatives may be used as biologically active compounds for the treatment of infections caused by these bacteria (especially citral, linalool, and geraniol). This, of course, requires the development of appropriate drug forms for the administration of these active substances, e.g., transdermal therapeutic systems. In the future, it is also worth examining the effects of mixtures of these compounds, not just individual components—perhaps this will increase the effectiveness of these compounds.

During our tests, in the case of bacteria of the *E. genus*, citral also showed the highest activity, but linalool, ocimenes, and geraniol showed a slightly lower activity. Enterococci are often the cause of urinary tract infections, postoperative and burn wounds, and endocarditis [85]. In recent years, enterococci have become an increasingly common cause of nosocomial infections. This may constitute a serious therapeutic problem due to their ability to form biofilms in wounds and the ability to acquire resistance to glycopeptide antibiotics, such as vancomycin, which are recommended for their treatment [85]. Our research results regarding the *Enterococcus* bacterial strains listed in Table 2 indicate that geraniol and its derivatives may be used as biologically active compounds capable of treating the infections caused by these bacteria (especially citral, linalool, ocimene, and geraniol). Perhaps in the future, the use of these compounds will help solve the problem of hospital infections, but this requires further research. One of the directions of research may be testing the biological activity of mixtures of these compounds.

**Figure 10:**
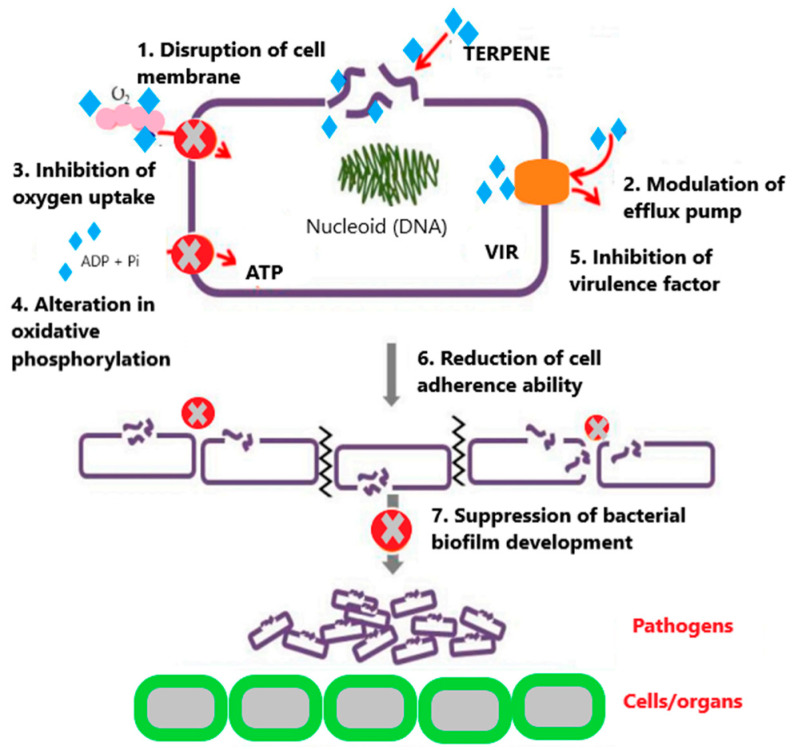
Mode of action of terpenes on antibiotic-resistant bacteria.

Griffin et al. [86] discovered in their research that most terpenes can hinder two crucial processes vital for the survival of microorganisms: oxygen uptake and oxidative phosphorylation. Oxygen is essential for the growth of aerobic microbes, as it provides the energy required for their development. On the other hand, oxidative phosphorylation is a fundamental biochemical process responsible for cellular respiration, occurring in the cytoplasmic membrane. Consequently, their interaction with terpenes induces a modification in cellular respiration, leading to the disconnection of oxidative phosphorylation in the microorganism [87]. Moreover, the carbonylation of terpenes has been demonstrated to enhance their bacteriostatic activity. A bacteriostatic substance impedes or inhibits the growth of microorganisms, while a bactericidal substance is accountable for the microorganism’s destruction. Terpenes have also displayed antiseptic potential, dependent on their solubility in water. The antimicrobial activity of terpenes is influenced by their lipophilicity and/or hydrophobicity, as well as the presence of hydroxyl groups [87]. In treatments related to the skin barrier, terpenes have been observed to impact lipid membrane activity. They interact with the lipophilic tails of intermembrane lipids and polar groups, thereby influencing lipoidal intermembrane and polar transmembrane pathways [88]. Although there are no specific reports on the modes of action of terpenes, a mechanism of action of phytochemicals found in nature has been proposed (Figure 10).

### 2.2. Microbiological Stability of Creams with Geraniol and Products of Its Transformation

**Figure 11:**
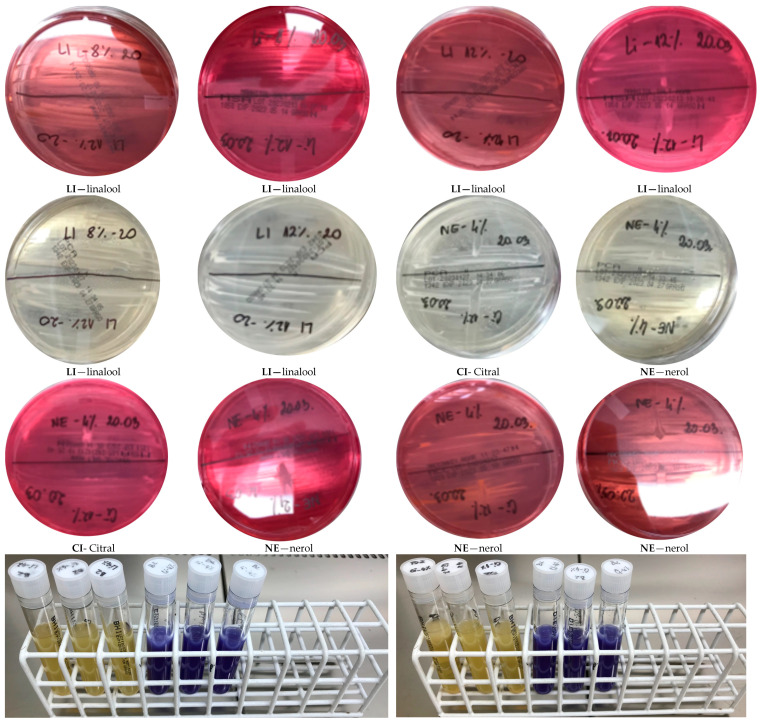
Example photos of the results obtained for creams with the addition of selected terpenes.

In tests of the microbiological purity of cream samples with various contents of geraniol and selected products of its transformation, in the range of 0.5%, 1.5%, 2.5%, 4%, 8%, and 12%, no growth of aerobic bacteria and fungi was found on any of the substrates used, both on plates with agar base (Table 2) and after multiplication in broths (Table 3), which proves the lack of microbiological contamination of the obtained cosmetic preparations and the microbiological stability of creams, even with a low percentage and weight content of the tested compounds (Figure 11). It should be assumed that the addition of the analyzed compounds had an influence on the durability of the creams. Moreover, it was observed that the addition of the tested compounds, even at concentrations of 4%, 8%, and 12%, had no effect on the change in the consistency of the cream samples and did not negatively affect the separation of phases during storage, and even had a positive effect on the organoleptic sensations by enriching the smell of the tested samples.

Both ointment bases and other components included in cosmetic preparations, which determine the proper permeability of medicinal substances, are a very good medium for the development of microorganisms. Microbiological purity determines the possibility of using the given preparation, as well as the duration of its use and storage. Bacteria and fungi that highly contaminate cosmetics contribute to the deterioration of their quality. Microorganisms multiplying in preparations during their use cause changes in their consistency, color, smell, separation of the cosmetic phases, or precipitation. Therefore, they limit the durability of the cosmetic preparation and even disqualify it from further use. Moreover, they can cause allergy, inflammation, and dermatoses [82].

To protect cosmetics, manufacturers use various types of preservatives. Properly selected preservatives cannot cause a therapeutic effect, but should be characterized by a good solubility, lack of toxicity, appropriate pH, and high antimicrobial activity. Moreover, they should not cause skin irritation, allergies, or other side effects. Therefore, the tested terpenes, due to their antimicrobial effect, are very promising substances for use as natural preservatives. Our research proves that the compounds used in the creams (geraniol and its derivatives) may be potential preservatives used in cosmetology.

## 3. Materials and Methods

### 3.1. Raw Materials, Cream Base, and Microbiological Media

In our studies we used geraniol (99%, Acros organics, Poznań, Poland), citral (95%, Sigma Aldrich, Poznań, Poland), beta-pinene (95%, Fluka, Poznań, Poland), nerol (97%, Acros organics, Poznań, Poland), ocimenes (90%, Sigma Aldrich), linalool (97%, Acros organics, Poznań, Poland), and farnesol (96% Acros organics, Poznań, Poland).

The nourishing base CALAYA (Cosibella, Leeds, UK), used for preparing natural cosmetics, contained aqua, *Helianthus annuus* (Sunflower) seed oil, Polysorbate 20, cetyl alcohol, glyceryl stearate, phenoxyethanol, carbomer, potassium sorbate, and sodium hydroxide.

Microbiological media used in experiments (Graso Biotech, Jabłowo, Poland) were as follows: Mueller–Hinton agar II, Columbia agar with 5% sheep blood, plate count agar, chocolate Agar, mannitol salt agar, BHI broth, and D/E neutralizing broth. Other reagents used in experiments included 0.9% sodium chloride solution (Polpharma, Starogard Gdański, Poland) and 70% ethanol (Chempur, Poznań, Poland).

### 3.2. Microorganisms and Inoculum

Gram-positive bacteria were used to determine the antimicrobial activity of geraniol and its derivatives, including two reference strains, *S. aureus* ATCC 29213 and *E. faecalis* ATCC 29212, as well as clinical strains with known drug sensitivity from the collection of the Department of Microbiology of the Multi-specialized Provincial Hospital in Gorzów Wielopolski: *S. epidermidis* MRSE, *S. aureus* MRSA, *E. faecalis* VRE VanB, *E. faecium* VRE VanA, and *E. faecium* VRE VanB.

### 3.3. Antibacterial Activity of Geraniol and Products of Its Transformation

The sensitivity of bacteria to geraniol and its derivatives was determined using the disc diffusion method used to determine the antimicrobial activity of antibiotics, which was adapted to the needs of the experiment, and was carried out in three repetitions. For this purpose, the strains were sown on solid media (Columbia agar with 5% sheep blood ans chocolate agar) and incubated at 37 °C, and then an inoculum of 0.5 on the McFarland scale was made from 18 h cultures by suspending bacterial colonies in a 0.9% solution of physiological saline using BioMerieux densimat. Suspensions of individual strains prepared in this way were inoculated on the surface using a sterile swab on Petri plates with Mueller–Hinton agar II or on Columbia agar containing 5% sheep blood. Five sterile paper discs with a diameter of 6 mm and soaked with 20 μL of the tested compounds were placed on the substrate surface. In total, 20 μL of 0.9% saline solution was used as a negative control, while the positive control was performed with 20 μL of 70% ethanol. The soaking time was 2 min. Plates prepared in this way were incubated for 24 h at 37 °C. After this time, the presence or absence of growth inhibition zones around the discs was checked. The diameter of the obtained growth inhibition zones was measured together with the disc diameter.

### 3.4. Preparation of Creams with Geraniol and Products of Its Transformation

This study used cream samples with different contents of geraniol and its transformation products (0.5%, 1%, 2.5%, 4%, 8%, and 12%). Table 4 shows the concentrations of geraniol and its transformation products expressed in mg/mL.

### 3.5. Microbiological Stability of Creams with Geraniol and Products of Its Transformation

The microbiological stability of the obtained creams was checked in two stages. In the first stage, the cream samples were inoculated onto solid and liquid culture media (PCA, mannitol salt agar, MacConkey agar, BHI broth, and D/E neutralizing broth) intended for the cultivation of aerobic bacteria and fungi using a sterile calibrated loop with a mesh size of 10 µL and were incubated at 37 °C for 24 h. After this time, the grown colonies of bacteria and fungi were checked on solid media and the results were given in CFU/mL (colony-forming units in 1 mL). In the second stage, liquid media were inoculated onto solid media using the sterile calibrated loop with a mesh size of 10 µL in order to verify the multiplication of minimal amounts of bacteria and fungi. After plating the broths onto solid media, the plates were again incubated at 37 °C for 24 h. Then, the grown colonies were checked. As the control, the cream base without the addition of the tested compounds was inoculated. During the experiment, the creams were stored at 4 °C. The experiment was carried out in three repetitions.

## 4. Conclusions

In our research, we carried out tests on the ability of geraniol and selected products of its transformation to combat seven different strains of Gram-positive bacteria. The sensitivity of the bacteria to geraniol and its derivatives was determined using the disc diffusion method, which is used to determine the antibacterial activity of antibiotics, which was adapted to the needs of the experiment, conducted in triplicate. The tested substances such as citral, linalool, nerol, and geraniol showed the greatest effectiveness against staphylococci, especially in the case of the *S. epidermidis* strain. For the remaining two *S. aureus* strains, both clinical and reference, a slightly lower effectiveness of the tested compounds was recorded. Only substances marked as β-pinene and farnesol did not show activity against all types of staphylococci tested. Our research results regarding the *Staphylococcus* bacterial strains indicate that geraniol and its derivatives (mainly citral, linalool, and nerol) may be biologically active compounds used for the treatment of infections caused by these bacteria. But these applications require the development of the appropriate drug forms for the administration of these active substances, e.g., transdermal therapeutic systems. In future, it is also worth examining the effects of mixtures of these compounds, not just individual components.

The second group of bacteria that was tested in our research is *E.* spp., for which, as in the case of *S.* spp., citral, linalool, geraniol, and, moreover, ocimenes showed the highest activity, while β-pinene and farnesol showed the lowest activity. Our research results regarding the *Enterococcus* bacterial strains showed that geraniol and its derivatives mentioned above, may be used as biologically active compounds capable of treating infections caused by these bacteria. Perhaps in the future, the use of these compounds will help to solve the problem of hospital infections, but this requires further research. The same as mentioned above, one of the directions of our future research may be testing the biological activity of mixtures of these compounds.

In our tests on the microbiological purity of cream samples with various geraniol contents and selected products of its transformation, we did not observe the growth of aerobic bacteria or fungi on any of the tested substrates. The creams we prepared were free from microbiological contaminants and were characterized by microbiological stability, even at low concentrations of the tested compounds. The addition of the tested compounds also had a positive effect on the durability of the creams and their smell. Moreover, even with higher contents of the tested compounds, the addition of these compounds did not change the consistency of the creams or cause phase separation during storage. The terpene compounds we tested are, therefore, very promising compounds that may be used as natural preservatives in the future.

To sum up, the obtained results indicate the wide possibilities of using the terpenes we tested in medicine and cosmetology. In the future, we plan to continue our research related to geraniol and its transformation products. We will perform tests on the stability of different types of creams prepared with these compounds and their potential cytotoxicity. In addition, an interesting aspect will be to test the ability of the studied terpenes to penetrate the skin, which may significantly expand the applications of these compounds, for example in transdermal delivery systems.

## Figures and Tables

**Table 1:** Zones of inhibition of the growth of selected Gram-positive bacteria around discs soaked with geraniol and its transformation products.

Compound/Bacteria	Zones of Inhibition [mm + SD]						
	*S. aureus* ATCC 29213	*S. aureus* MRSA	*S. epidermidis* MRSE	*E. faecalis* ATCC 29212	*E. faecalis* VRE VanB	*E. faecium* VRE VanA	*E. faecium* VRE VanB
	[mm]	[mm]	[mm]	[mm]	[mm]	[mm]	[mm]
Control negative (0.9% NaCl)	6 ± 0	6 ± 0	6 ± 0	6 ± 0	6 ± 0	6 ± 0	6 ± 0
Control positive (70% ethanol)	15 ± 0	14 ± 0	15 ± 0	13 ± 0	13 ± 0	13 ± 0	13 ± 0
Geraniol (GE)	11 ± 0	17.4 ± 0.5	17.4 ± 0.5	6 ± 0	9.8 ± 0.4	20.2 ± 0.4	12 ± 0
Citral (CI)	39.6 ± 0.5	20 ± 0	42.6 ± 0.5	36.8 ± 0.8	14.6 ± 0.5	40 ± 0	39.6 ± 0.5
β-pinene (BP)	6 ± 0	6 ± 0	6 ± 0	6 ± 0	6 ± 0	6 ± 0	6 ± 0
Nerol (NE)	12.4 ± 0.5	15.6 ± 0.5	18.6 ± 0.5	9.8 ± 0.4	11.4 ± 0.5	16.2 ± 0.4	16.2 ± 0.4
Farnesol (FA)	6 ± 0	6 ± 0	6 ± 0	6 ± 0	6 ± 0	10 ± 0	10 ± 0
Linalool (LI)	40 ± 0	23.6 ± 0.5	30 ± 0	23.8 ± 0.4	25 ± 0	14 ± 0	37.8 ± 0.4
Ocimenes (OC)	12 ± 0	10 ± 0	10 ± 0	15.4 ± 0.5	10 ± 0	12 ± 0	9.8 ± 0.4
the diameter of the sterile disc was 6 mm							

**Table 2:** Results of bacteriological inoculation of cream samples with various percentages of geraniol and its transformation products on MC, PCA, and MSA solid media.

CREAM BASE + COMPOUND		PCA	MC	MSA
Cream base (BZ)		0 CFU/mL	0 CFU/mL	0 CFU/mL
Geraniol (GE)	0.5%	0 CFU/mL	0 CFU/mL	0 CFU/mL
	1.5%	0 CFU/mL	0 CFU/mL	0 CFU/mL
	2.5%	0 CFU/mL	0 CFU/mL	0 CFU/mL
	4%	0 CFU/mL	0 CFU/mL	0 CFU/mL
	8%	0 CFU/mL	0 CFU/mL	0 CFU/mL
	12%	0 CFU/mL	0 CFU/mL	0 CFU/mL
Citral (CI)	0.5%	0 CFU/mL	0 CFU/mL	0 CFU/mL
	1.5%	0 CFU/mL	0 CFU/mL	0 CFU/mL
	2.5%	0 CFU/mL	0 CFU/mL	0 CFU/mL
	4%	0 CFU/mL	0 CFU/mL	0 CFU/mL
	8%	0 CFU/mL	0 CFU/mL	0 CFU/mL
	12%	0 CFU/mL	0 CFU/mL	0 CFU/mL
β-pinen (BP)	0.5%	0 CFU/mL	0 CFU/mL	0 CFU/mL
	1.5%	0 CFU/mL	0 CFU/mL	0 CFU/mL
	2.5%	0 CFU/mL	0 CFU/mL	0 CFU/mL
	4%	0 CFU/mL	0 CFU/mL	0 CFU/mL
	8%	0 CFU/mL	0 CFU/mL	0 CFU/mL
	12%	0 CFU/mL	0 CFU/mL	0 CFU/mL
Nerol (NE)	0.5%	0 CFU/mL	0 CFU/mL	0 CFU/mL
	1.5%	0 CFU/mL	0 CFU/mL	0 CFU/mL
	2.5%	0 CFU/mL	0 CFU/mL	0 CFU/mL
	4%	0 CFU/mL	0 CFU/mL	0 CFU/mL
	8%	0 CFU/mL	0 CFU/mL	0 CFU/mL
	12%	0 CFU/mL	0 CFU/mL	0 CFU/mL
Linalool (LI)	0.5%	0 CFU/mL	0 CFU/mL	0 CFU/mL
	1.5%	0 CFU/mL	0 CFU/mL	0 CFU/mL
	2.5%	0 CFU/mL	0 CFU/mL	0 CFU/mL
	4%	0 CFU/mL	0 CFU/mL	0 CFU/mL
	8%	0 CFU/mL	0 CFU/mL	0 CFU/mL
	12%	0 CFU/mL	0 CFU/mL	0 CFU/mL
Farnesol (FA)	0.5%	0 CFU/mL	0 CFU/mL	0 CFU/mL
	1.5%	0 CFU/mL	0 CFU/mL	0 CFU/mL
	2.5%	0 CFU/mL	0 CFU/mL	0 CFU/mL
	4%	0 CFU/mL	0 CFU/mL	0 CFU/mL
	8%	0 CFU/mL	0 CFU/mL	0 CFU/mL
	12%	0 CFU/mL	0 CFU/mL	0 CFU/mL
Ocimenes (OC)	0.5%	0 CFU/mL	0 CFU/mL	0 CFU/mL
	1.5%	0 CFU/mL	0 CFU/mL	0 CFU/mL
	2.5%	0 CFU/mL	0 CFU/mL	0 CFU/mL
	4%	0 CFU/mL	0 CFU/mL	0 CFU/mL
	8%	0 CFU/mL	0 CFU/mL	0 CFU/mL
	12%	0 CFU/mL	0 CFU/mL	0 CFU/mL

**Table 3:** Results of bacteriological inoculation of cream samples with various percentages of geraniol and its transformation products propagated on BHI broth and D/E neutralizing broth liquid media and after sowing on MC, PCA, and MSA solid media.

CREAM BASE + COMPOUND		BHI Broth	D/E Neutralizing Broth
Cream base (BZ)		0 CFU/mL	0 CFU/mL
Geraniol (GE)	0.5%	0 CFU/mL	0 CFU/mL
	1.5%	0 CFU/mL	0 CFU/mL
	2.5%	0 CFU/mL	0 CFU/mL
	4%	0 CFU/mL	0 CFU/mL
	8%	0 CFU/mL	0 CFU/mL
	12%	0 CFU/mL	0 CFU/mL
Citral (CI)	0.5%	0 CFU/mL	0 CFU/mL
	1.5%	0 CFU/mL	0 CFU/mL
	2.5%	0 CFU/mL	0 CFU/mL
	4%	0 CFU/mL	0 CFU/mL
	8%	0 CFU/mL	0 CFU/mL
	12%	0 CFU/mL	0 CFU/mL
β-pinen (BP)	0.5%	0 CFU/mL	0 CFU/mL
	1.5%	0 CFU/mL	0 CFU/mL
	2.5%	0 CFU/mL	0 CFU/mL
	4%	0 CFU/mL	0 CFU/mL
	8%	0 CFU/mL	0 CFU/mL
	12%	0 CFU/mL	0 CFU/mL
Nerol (NE)	0.5%	0 CFU/mL	0 CFU/mL
	1.5%	0 CFU/mL	0 CFU/mL
	2.5%	0 CFU/mL	0 CFU/mL
	4%	0 CFU/mL	0 CFU/mL
	8%	0 CFU/mL	0 CFU/mL
	12%	0 CFU/mL	0 CFU/mL
Linalool (LI)	0.5%	0 CFU/mL	0 CFU/mL
	1.5%	0 CFU/mL	0 CFU/mL
	2.5%	0 CFU/mL	0 CFU/mL
	4%	0 CFU/mL	0 CFU/mL
	8%	0 CFU/mL	0 CFU/mL
	12%	0 CFU/mL	0 CFU/mL
Farnesol (FA)	0.5%	0 CFU/mL	0 CFU/mL
	1.5%	0 CFU/mL	0 CFU/mL
	2.5%	0 CFU/mL	0 CFU/mL
	4%	0 CFU/mL	0 CFU/mL
	8%	0 CFU/mL	0 CFU/mL
	12%	0 CFU/mL	0 CFU/mL
Ocimenes (OC)	0.5%	0 CFU/mL	0 CFU/mL
	1.5%	0 CFU/mL	0 CFU/mL
	2.5%	0 CFU/mL	0 CFU/mL
	4%	0 CFU/mL	0 CFU/mL
	8%	0 CFU/mL	0 CFU/mL
	12%	0 CFU/mL	0 CFU/mL

**Table 4:** The concentrations of geraniol and its transformation products expressed in mg/mL.

Concentration	0.5%	1.5%	2.5%	4%	8%	12%
	mg/mL	mg/mL	mg/mL	mg/mL	mg/mL	mg/mL
Geraniol (GE)	4.45	13.35	22.25	35.6	71.2	106.8
Citral (CI)	4.45	13.35	22.25	35.6	71.2	106.8
β-pinene (BP)	4.3	12.9	21.5	34.4	68.8	103.2
Nerol (NE)	4.45	13.35	22.25	35.6	71.2	106.8
Farnesol (FA)	4.45	13.35	22.25	35.6	71.2	106.8
Linalool (LI)	4.3	12.9	21.5	34.4	68.8	103.2
Ocimenes (OC)	4.3	12.9	21.5	34.4	68.8	103.2

## Data Availability

The data presented in this study are available on request from the corresponding authors.
